# The implications of weeklong fostering and co-housing on shelter dog welfare

**DOI:** 10.7717/peerj.20608

**Published:** 2026-01-27

**Authors:** Lisa M. Gunter, JoAnna M. Platzer, Jenifer L. Reed, Emily M. Blade, Rachel J. Gilchrist, Rebecca T. Barber, Erica N. Feuerbacher, Clive D.L. Wynne

**Affiliations:** 1 School of Animal Sciences, Virginia Polytechnic Institute and State University (Virginia Tech), Blacksburg, VA, United States of America; 2 Department of Psychology, Arizona State University, Tempe, AZ, United States of America; 3 Office of Financial Planning and Budget, Michigan State University, East Lansing, MI, United States of America

**Keywords:** Dogs, Animal shelter, Welfare, Human-animal interaction, Housing, Conspecific, Enrichment, Cortisol, Activity

## Abstract

Meeting the needs of dogs in a typical animal shelter can be a challenging proposition. Negative environmental inputs, such as excessive noise, restrictive kenneling, and social isolation, contribute to the compromised welfare that dogs experience. Human-animal interaction, such as a temporary stay outside of the shelter in a caregiver’s home, has been shown to reduce dogs’ cortisol levels and increase their rest. What is less understood is if longer durations of foster care could extend those benefits. In addition, dogs living with a conspecific in the shelter, co-housing, has been even less explored, but available findings suggest that dogs’ behavior can be improved by living with another dog. In the present study, we investigated the impacts of weeklong fostering on dogs’ urinary cortisol and activity. Two animal shelters, one open and one managed admission, participated. Exclusively at the open admission facility, a smaller sub-study explored the effects of co-housing prior to foster care (*i.e.*, with and without a dog) and following (*i.e.*, without another dog or with a familiar or new dog) in the animal shelter. To answer these research questions, dogs’ urine was collected in the morning for cortisol: creatinine analysis and activity monitors were worn by the dogs for 17 days: five days in the animal shelter, seven days in a caregiver’s home, and five days in the shelter following foster care. In total, 84 dogs participated with 1,385 cortisol:creatinine values and 1,205 activity totals across five activity level types. At both shelters, we found dogs’ cortisol levels decreased, and they spent more time resting during weeklong fostering. Moreover, no significant differences in cortisol or activity were found pre- and post-fostering, with the exception of more time being spent in mid-intensity activity in the shelter following foster care as compared to before. These findings align with investigations of shorter durations of foster care, although the magnitude of the present intervention’s impact was greater. With regards to the type of housing dogs experienced (with or without another dog), no difference was found in dogs’ cortisol values in either the days before or after foster care with no effect on their activity detected pre-fostering; however, dogs’ activity was influenced by living with a familiar dog upon reentry to the animal shelter following foster care. Specifically, dogs rested more and engaged in less high activity, indicating a positive effect on their welfare. Lastly as has been previously observed, significant differences in cortisol and activity were found between our shelters, suggesting that environmental differences are contributing to canine welfare that require further scientific exploration. In total, a weeklong reprieve from the animal shelter, as well as co-housing with a familiar dog upon return to the shelter are two evidence-based interventions that can improve the welfare of shelter-living dogs.

## Introduction

Millions of dogs enter animal shelters each year in the United States (ASPCA, 2025). Once dogs arrive at an animal shelter, most will leave alive: be it direct rehoming to a new adopter, transferring to another agency for placement, or reuniting with their owner; euthanasia comprises a relatively small proportion of outcomes for shelter dogs (Woodruff et al., 2021; Woodruff & Smith, 2020). One type of intervention employed by animal shelters, the fostering of dogs in homes by temporary caregivers, has been shown to increase dogs’ likelihood of adoption when compared to dogs that do not receive such experiences (Gunter et al., 2023; Patronek & Crowe, 2018).

Utilizing Mellor et al.’s (2020) Five Domains Model, it is likely that negative inputs related to the physical environment as well as social isolation from other animals and humans compromise the welfare of dogs living in animal shelters. Previous research has shown that noise levels can exceed decibel limits set by the Occupational Safety and Health Administration for unprotected human exposure during an eight-hour period, indicating that these environments may be harmful, at times, to people and dogs (Sales et al., 1997; Coppola, Enns & Grandin, 2006; Scheifele et al., 2012; Venn, 2013). Moreover, kenneling in the shelter inhibits dogs’ natural movements, reduces their opportunities for spontaneous activity, and provides limited predictability and control (Hubrecht et al., 1995; Hennessy et al., 1998).

One measure that informs our understanding of dogs’ experience in the animal shelter is the hormone cortisol (Hewson, Hiby & Bradshaw, 2007). Research by Sandri et al. (2015) and van der Laan et al. (2021; 2023a) has found that the cortisol levels of shelter-living dogs are higher than those of owned dogs, most notably at intake into the shelter (Hennessy et al., 1997). Within-subject-designed studies have also detected elevated cortisol levels when dogs are residing in kennels as compared to homes (Rooney, Gaines & Bradshaw, 2007; Fehringer, 2014; Gunter et al., 2019; Van der Laan et al., 2021; Van der Laan, Vinke & Arndt, 2022; Van der Laan, Vinke & Arndt, 2023a; Van der Laan, Vinke & Arndt, 2023b). Utilizing hair cortisol concentrations, van der Laan and colleagues (2022) found that dogs’ cortisol levels did not significantly differ at intake (*i.e.,* reflecting their pre-shelter experience) and after adoption, while concentrations obtained in the shelter were higher than any other time point.

Measuring activity can further aid our assessment of dogs’ welfare. Multiple studies have found that the sleeping, resting, and activity behaviors of dogs differ when living in the animal shelter (Gunter et al., 2019; Hoffman, Ladha & Wilcox, 2019; Van der Laan et al., 2021; Van der Laan, Vinke & Arndt, 2023a; Van der Laan, Vinke & Arndt, 2023b). Compared to dogs in homes, shelter-residing dogs are more active during both their most and least active hours (Hoffman, Ladha & Wilcox, 2019), and rest less at night (Van der Laan et al., 2021). This observed difference in rest is particularly evident in dogs’ first two nights in the shelter and persists nearly two weeks later, although dogs are resting more later in their stay (Van der Laan et al., 2021). When shelter dogs sleep more during the daytime, they are more optimistic and emit fewer repetitive behaviors in the kennel (Owczarczak-Garstecka & Burman, 2016).

In the US, dogs are often singly housed in shelters to reduce the spread of diseases, injury between dogs, and difficulty monitoring individual food and water intake (DeTar et al., 2022). Nevertheless, group housing has been shown to decrease the frequency of dogs’ repetitive behaviors as compared to single housing, allowing for conspecific interactions with relatively low incidences of injury (Hubrecht, Serpell & Poole, 1992; Mertens & Unshelm, 1996). Dalla Villa et al. (2013) observed that resting behavior, as indicated by lying down, increased when dogs were housed in pairs *versus* groups, while Grigg et al. (2017) found few differences in dogs’ behavior when kenneled alone as compared to living with another dog. A more recent study by Hecker et al. (2024) detected that dogs lip-licked, whined, and had their ears back less often when pair-housed *versus* singly in the shelter, and had shorter lengths of stay awaiting adoption.

Across multiple studies with shelter-living dogs, time spent with a person outside of the kennel reduces measures of stress and improves behavior; and as such, is probably the most beneficial type of social interaction (Gunter & Feuerbacher, 2022). Furthermore, one, two, and three days of foster care in a home, as described by Gunter et al. (2019) and Fehringer (2014), have been shown to reduce dogs’ cortisol levels, although two-and-a-half hour outings into the community with a person increase dogs’ cortisol levels, even after accounting for their increased activity (Gunter et al., 2021). Dogs’ rest also improves when they leave the shelter. Gunter et al. (2019) found that dogs had their longest bouts of interrupted rest during a two-night fostering stay, as compared to the shelter; and similarly, Van der Laan, Vinke & Arndt (2023b) found that dogs’ nocturnal activity decreased two nights following adoption.

While the effects of temporary fostering on shelter dog welfare have been previously explored, what is less understood is whether dogs experience greater benefits during longer durations of foster care as well as negative impacts on their cortisol or activity levels upon return to the animal shelter. When Gunter et al. (2019) examined the effects of one and two nights of fostering, they found that dogs’ cortisol increased and resting decreased upon return to the shelter. While these post-intervention levels were not statistically higher than pre-intervention (and resting bouts were still longer in the shelter following foster care than prior), it is unknown whether cortisol and activity levels would continue to rise over subsequent days of observation. Previously, Hiby, Rooney & Bradshaw (2006) found that dogs’ urinary cortisol levels, when measured on their first, second, third, fifth, seventh, and tenth days following intake into the shelter, increased for dogs that were relinquished by their owners, while dogs that had been returned after a failed adoption or brought to the animal shelter as a stray showed decreasing cortisol levels.

In the present study, we hypothesized that fostering for seven days would result in improved welfare for dogs awaiting adoption as demonstrated by lower urinary cortisol:creatinine (C/C) levels and greater proportions of time spent resting in the foster home as compared to time points in the shelter, either before or after fostering. Additionally, we explored the impacts of co-housing in a smaller sub-study within our larger investigation. We hypothesized that dogs that were housed with another dog would have lower C/C ratios and spend more time resting compared to dogs housed alone. Furthermore, upon return from foster care, we predicted that dogs that were co-housed with the same dog that they had lived with prior to fostering would have lower C/C ratios than dogs that were kenneled with a new dog or living alone.

## Materials & Methods

### Shelters

Data were collected at two animal shelters in the United States: Pima Animal Care Center in Tucson, AZ (PACC, July–August 2021), and Charlottesville-Albemarle Society for the Prevention of Cruelty to Animals in Charlottesville, VA (CASPCA, November 2021–January 2022). PACC is an open admission, municipal animal shelter, and CASPCA is a managed admission, non-profit animal shelter with municipal contracts in the surrounding area. Annual canine intake for 2021 for PACC and CASPCA was 10,523 and 1,750 dogs, respectively.

Staff at these shelters determined which dogs and foster caregivers participated in the study. Veterinary staff evaluated dogs’ health prior to participation, although it is possible that dogs with underlying diseases without overt symptoms were included in our sample. Additionally, researchers further selected dogs without observed aggressive behavior towards people or other dogs. Dogs that were fearful of the urine collection equipment were excluded from the study if researchers were unable to collect urine samples for cortisol:creatinine analysis.

### Housing

In the housing sub-study carried out at PACC, dogs in the shelter either lived with another dog (co-housed) or by themselves (singly housed). Both housing arrangements occurred at PACC prior to this study, although at the time co-housing was more common due to capacity constraints.

To determine dogs’ suitability for co-housing, shelter staff and members of the research team conducted introductions between dogs in outdoor fenced yards at the shelter. Introductions began on-leash and graduated to off-leash interactions if the dogs were compatible. Dogs’ behavior was assessed during these interactions, including but not limited to proximity-seeking by both dogs, relaxed body language, and a lack of aggressive behavior (*i.e.,* barking, growling, lunging). Those with compatible interactions off-leash were housed together.

Prior to fostering, dogs were categorized as either living singly or co-housed in the shelter. Following foster care, dogs either lived by themselves, with the dog they were co-housed with prior to fostering, or co-housed with a new dog.

### Fostering

Shelters provided caregivers foster training, and researchers described the study and supplied urine collection equipment. If dogs were to be fostered in homes with other dogs, dogs were introduced at the shelter prior to fostering and deemed compatible utilizing the same behaviors described for co-housing. If dogs were incompatible once in a caregiver’s home, resident and fostered dogs were separated or the fostered dog was returned to the shelter, which rarely occurred. Separation instructions were provided for households with cats.

Dogs were fostered in caregiver homes for seven days with the research team remaining in contact with them daily. When dogs returned to the shelter after the fostering period, the research team met with the caregivers, collected the dogs, their urine samples and equipment, and returned the dogs to kennels in the shelter.

### Urine collection

Dogs were enrolled for a total of 17 days across three phases of the study: five days in the shelter, seven days in foster care, and five days post-fostering in the animal shelter. Urine collection occurred each morning between 7:00 am and 9:30 am with the time of collection recorded for each sample. The research team collected samples at the shelter while foster caregivers did so when the dogs were living in their homes. Urine collection was utilized as a non-invasive means of biological collection for the measurement of cortisol with shelter dogs and is consistent with the methods described by Gunter et al. (2019) and Gunter et al. (2021). A total of 3.39% of samples were collected outside of this window. When dogs did not readily urinate, they were provided a mixture of wet food and water and were walked again until they urinated resulting in a sample collected later than the described window (2.02% of samples). Additionally, a small number of fostered dogs urinated earlier than 7:00 am (1.37% of samples).

Samples were collected in Olympic Clean-Catch disposable plates taped to the grasping end of 91-cm “Pickup and Reach” tool (Harbor Freight, Calabasas, CA). Urine was poured from collection trays into five mL plastic vials for storage and future analysis. Post collection, trays were rinsed with water and air dried or wiped with KimWipes (Kimberly-Clark, Irving, TX, USA). Urine samples were promptly placed in a portable cooler with ice, and within two hours of collection into a residential freezer at a temperature of approximately −18 °C.

As previously described by Gunter et al. (2021), frozen urine samples were shipped overnight on dry ice to Zoetis Reference Laboratories (Louisville, KY, USA) for C/C analysis. Analysis was conducted using an automated wet biochemistry analyzer (AU680; Beckman Coulter, Brea, CA, USA) for measurement of creatinine. Bio-Rad Liquid Human Urine Precision Chemistry Controls 1 and 2 (Control Level 1 #397, Control Level 2 #398; Bio-Rad Laboratories, Inc., Hercules, CA, USA) were run during urine sample testing and stored according to manufacturer instructions. Cortisol was measured using a commercially available product designed for an enzyme-amplified chemiluminescence assay system (Immulite 2000 XPi; Siemens Healthcare Diagnostics, Inc., Newark, DE, USA). Cortisol:creatinine ratios (measured in µmol/L:µmol/L) ×10^−6^ were then calculated.

### Activity monitoring

Whistle FIT activity monitors (Whistle Labs Inc., San Francisco, CA, USA) were attached to dog collars, allowing for collection of their movement. These collar-mounted monitors were placed on the dogs in the afternoon prior to dogs’ first day of urine collection. Given the duration of dogs’ participation in the study, more than one monitor was used. New monitors were typically placed on dogs prior to entering foster care and upon return to the animal shelter, although unexpected battery loss or damage necessitated additional removals and replacements. Data from Whistle FIT monitors were transmitted to Whistle servers *via* wireless networks (in the shelter and foster home) and Bluetooth.

The procedure for calculating activity was first described in Gunter et al. (2021) and will be briefly described here. Dogs’ activity was calculated by using the raw data generated by the Whistle FIT monitors. These triaxial accelerometers collected the x, y, and z components of vectors representing dogs’ movements at a rate of up to 50 times per minute. Magnitudes of these vectors were then calculated as indications of dogs’ composite movements across each minute. Magnitude calculations were summed over one-minute epochs as an estimate of a dog’s activity within that minute. In this study, magnitude-per-minute (m/m) values ranged from 0.79 to 9,578.68.

M/m values were categorized into one of five, evenly apportioned activity levels. These quintile thresholds were derived from all m/m values obtained during the study with each quintile containing approximately 210,136 records. Magnitude-per-minute thresholds for each activity level are shown in Table 1. These values are similar to the thresholds utilized in Gunter et al. (2021).

Dogs’ total minutes in each activity level were calculated for the three study phases: in the shelter prior to the intervention, during the dog’s time in foster care, and in the shelter after the intervention.

### Statistical analysis

To understand the impact of the weeklong fostering intervention, we carried out statistical analyses on dogs’ cortisol and activity levels. With dogs’ C/C ratios, we used repeated measures linear mixed models that explored cortisol levels in two ways: across the 17 days of the study and then by phase: (1) before, (2) during, and (3) after foster care. This allowed us to investigate whether cortisol differed across time (at the day or phase level), by shelter, or in a shelter-by-time interaction.

In order to examine whether dogs’ activity differed across the phases of the study, by level or shelter, we used a generalized linear model. Dogs’ activity in each of five levels was calculated for each of the three study phases. In this model, main effects of activity level, phase, and shelter; two-way interactions between activity level and phase, shelter and activity level, and shelter and phase; and a three-way interaction of shelter, activity level, and phase were included as fixed effects.

To understand the impact of housing on dogs’ welfare, we carried out analyses on their cortisol and activity levels. Utilizing dogs’ C/C ratios, we employed two repeated measures linear mixed models, pre- and post-foster care (Days 1–5 and 13–17, respectively). Dog and intercept were entered as random effects with housing, study day, and the housing-by-day interaction as fixed effects with other dog variables retained according to model fit. The type of housing that dogs experienced was categorized as: single, co-housing between two dogs that had been housed together previously (familiar), or co-housing with a new dog (new).

**Table 1:** Magnitude-per-minute thresholds for each quintile of activity level derived from raw data collected by dogs’ Whistle FIT activity monitors.

** Activity level**	**Threshold**	
	Low	High
Q1 (Lowest)	0.79	733.77
Q2	733.78	1,656.09
Q3	1,656.10	2,746.70
Q4	2,746.71	3,076.01
Q5 (Highest)	3,076.02	9,578.68

To examine whether time dogs spent in the five activity levels was influenced by housing type, we employed two generalized linear models: one that utilized dogs’ activity in the shelter prior to foster care (phase one) and another that examined their activity after foster care (phase three). As with the housing analyses described above that explored dogs’ cortisol values, housing was described as either single or co-housing with a familiar or new dog. Activity level, housing, and a housing-by-activity level interaction were entered as main effects into these models with no additional covariates.

For our mixed models, an identity covariance matrix and a first-order autoregressive structure with heterogenous variances for the repeated measure of time were used. The method of Restricted Maximum Likelihood (REML) was employed to estimate parameter values with the lowest Bayesian Information Criterion (BIC) value used to indicate best fit. For our generalized linear models, binomial probability distributions and logit link functions were used. When *post-hoc* comparisons were conducted in our weeklong fostering analyses, Sidak corrections were used to reduce the likelihood of false positives when multiple comparisons were made. In our housing analyses, in consideration of the smaller sample size and exploratory nature of the research question, Least Significant Difference tests were used. A statistical significance level of *p* <0.05 was applied throughout all models.

### Ethical statement

Procedures carried out at PACC and CASPCA were approved by the Arizona State University Institutional Animal Care and Use Committee (IACUC: 20–1760R).

## Results

### Descriptive statistics

A total of 84 dogs participated in this study, 42 at each shelter. Descriptive statistics about the dogs are included in Table 2. Overall, 81 weeklong fostering experiences were carried out as part of this study, with 39 dogs fostered at PACC and 42 dogs at CASPCA.

**Table 2:** Descriptive statistics about participating dogs at two animal shelters, PACC and CASPCA.

**Variable**	**PACC**	**CASPCA**	**Overall sample**
Intake type			
% Stray	59.5	7.1	33.3
% Owner surrender & Return	35.7	0	17.9
% Cruelty/confiscate	4.8	0	2.4
% Transfer-in	0	92.9	46.4
Sex (% Male)	52.4	47.6	50.0
*M* Age (months)	52.6	25.6	39.1
	*36.8*	*18.7*	*32.0*
*M* Weight (kg)	27.8	20.0	23.9
	*5.9*	*6.4*	*7.2*
*M* Length of stay (days)	25.3	8.1	16.7
	*27.4*	*5.6*	*21.5*

**Note.**

Values in italics underneath *M* age, weight, and length of stay are standard deviations.

At PACC, 12 dogs were singly housed, and 30 were co-housed prior to foster care (12 were with another dog in the study). Nine co-housed dogs living with another dog were singly housed for their first day of urine collection and then transitioned to co-housing for the remaining four days. Following foster care, 14 dogs were singly housed, eight were co-housed with a familiar dog that they were living with prior to fostering (that was also in the study), and 17 dogs were co-housed with a new dog. A total of two co-housed dogs living with a new dog were singly housed on their last day of the study due to the adoption of their kennelmate.

### Weeklong fostering: cortisol analyses

#### By day

Urine collections at PACC and CASPCA yielded a total of 1,385 cortisol:creatinine values that were utilized in our statistical analyses of the fostering intervention. We analyzed these values across the study’s 17 days using a linear mixed model to detect an effect of day, shelter, or shelter-by-day interaction with dogs’ sex, weight, age, and (log) LOS. Dogs’ weight and age were not retained in the model as their removal led to a better model fit as determined by BIC and described in Table 3.

The main effect of day was significant, *F* (16, 252.77) = 11.10, *p* < .001, indicating that dogs’ cortisol levels changed across the 17 days of our study. In *post-hoc* comparisons, dogs had significantly lower cortisol values during their stay in a foster home, particularly Days 7–12, in comparison to their time in the shelter prior to (Days 1–5) and after (Days 13–17) foster care. Dogs’ first day of fostering (Day 6 of the study) was the highest cortisol value measured in the home and was not significantly different than any day in the shelter, pre- or post-fostering. Day 10, the dogs’ fifth day of fostering (which had the most variability of any day in foster care), was only significantly lower than Days 1 and 3 in the shelter, marginally so with Day 4 (*p* = .064), and Days 14–17 in the shelter following foster care. C/C ratios in the shelter, before and after fostering, did not significantly vary in *post-hoc* comparisons.

**Table 3:** Best-fitting linear mixed model predicting dogs’ daily cortisol levels.

**Source**	*Numerator df*	*Denominator df*	*F*	*p*
Intercept	1	120.93	45.25	<.001
Day	16	252.77	11.10	<.001
Shelter	1	93.84	14.77	<.001
Day * Shelter	16	235.91	1.28	.211
Sex	1	78.35	0.60	.807
(log) LOS	1	122.25	1.31	.254

**Figure 1:**
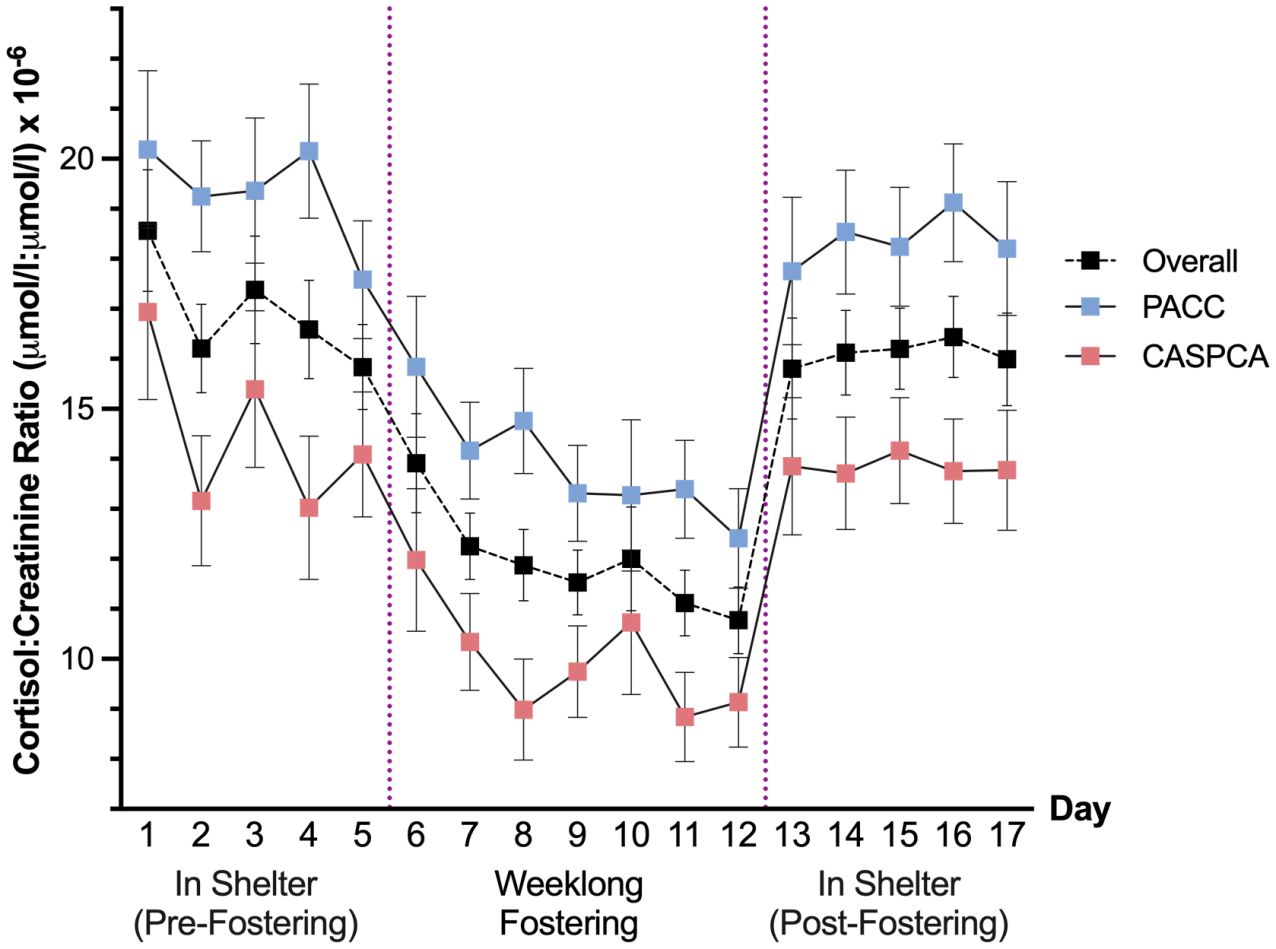
Estimated marginal means and standard errors of dogs’ urinary cortisol values across 17 days overall and at PACC and CASPCA.

A main effect of shelter was also detected, *F* (1, 93.84) = 14.77, *p* < .001, signifying that cortisol values at our two shelters differed. Specifically, dogs at CASPCA had significantly lower estimated marginal means for their C/C ratios (12.45) than dogs at PACC (16.80). Figure 1 presents the estimated marginal means and standard errors of the cortisol values for the 17 days of the study at each shelter and overall.

#### By study phase

To better understand the fostering intervention’s overall impact, we subsequently analyzed these mean cortisol values across the three study phases using a linear mixed model to detect an effect of phase, shelter, or shelter-by-phase interaction with dogs’ sex, weight, age, and (log) average LOS added as covariates. The variables of age and weight were removed to improve model fit. The final model, as determined by the smallest BIC value, is displayed in Table 4.

The main effect of phase was significant, *F* (2, 109.79) = 34.76, *p* < .001, indicating that dogs’ average cortisol levels differed across the study phases. In *post-hoc* comparisons, dogs had significantly lower average C/C values during weeklong fostering as compared to their time in the shelter before (*p* < .001; d_av_ = 0.81) or after (*p* < .001; d_av_ = 0.68) foster care, and no difference was found in their average cortisol levels between in-shelter phases (*p* = .659).

**Figure 2:**
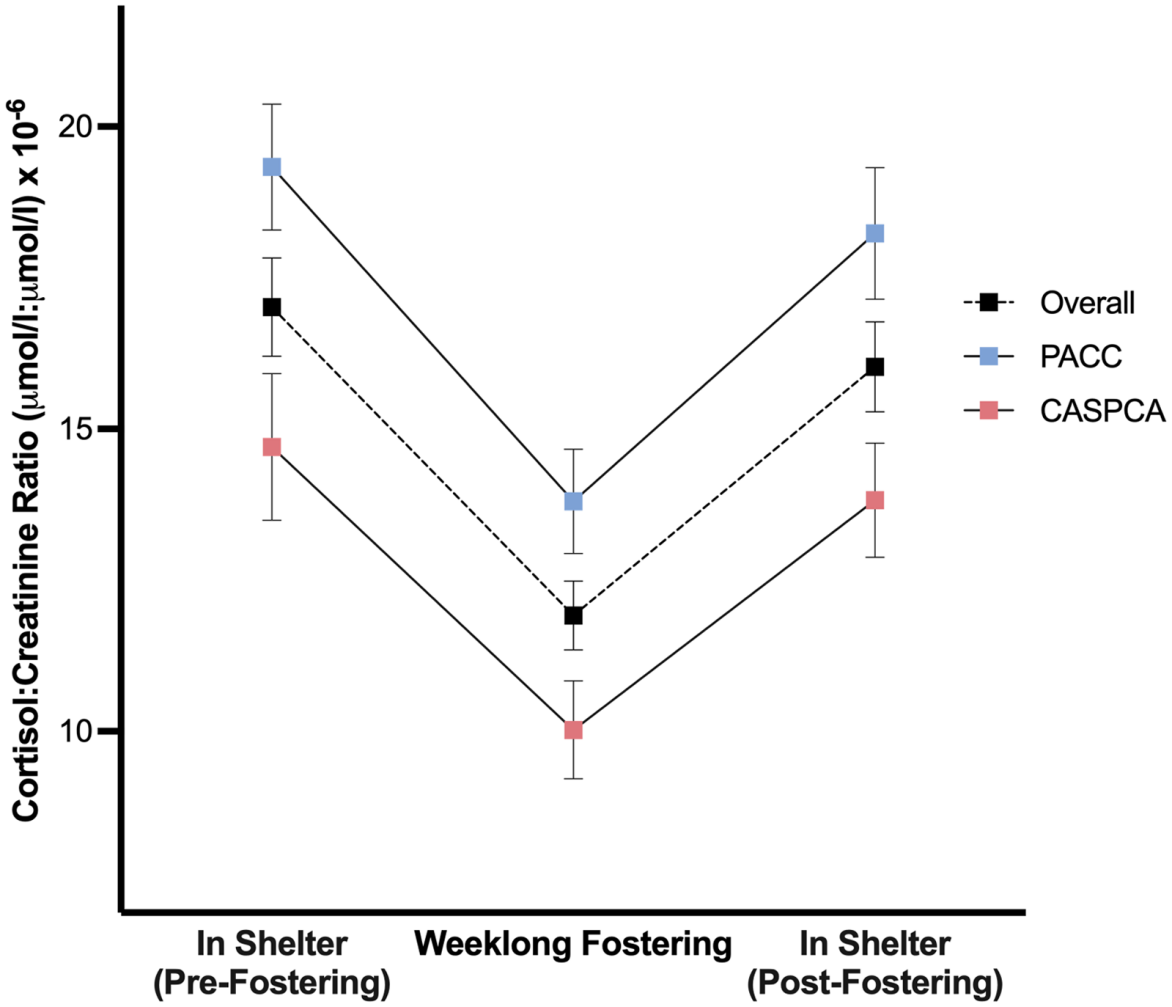
Estimated marginal means and standard errors of dogs’ average urinary cortisol values during each of the study’s phases overall and at PACC and CASPCA.

A main effect of shelter was also found, *F* (1, 84.05) = 13.19, *p* < .001, d_av_ = 0.83, signifying that the average cortisol values at our two shelters differed. Particularly, dogs’ average C/C ratios were lower at CASPCA (12.85) than dogs at PACC (17.12). Figure 2 presents the estimated marginal means and standard errors of the average cortisol values for each of the study phases at PACC, CASPCA, and overall.

### Weeklong fostering: activity analysis

#### By phase

At PACC and CASPCA, 84 dogs provided 1,205 totals (in hours) for the five activity levels across the three phases of the study: in-shelter pre-fostering, during weeklong fostering, and in-shelter post-fostering. Our statistical model included the fixed effects of activity level (Q1–Q5), phase (1–3), and shelter (PACC and CASPCA). Interactions of phase-by-activity-level, shelter-by-activity-level, shelter-by-phase, and phase-by-activity-level-by-shelter were also entered into model. Most main effects and interactions were statistically significant (Table 5).

**Table 4:** Best-fitting linear mixed model predicting dogs’ phase cortisol levels.

**Source**	*Numerator df*	*Denominator df*	*F*	*p*
Intercept	1	107.45	37.93	<.001
Phase	2	109.79	34.76	<.001
Shelter	1	84.05	13.19	<.001
Phase * Shelter	2	82.30	0.23	.796
Sex	1	77.91	0.01	.943
(log) Average LOS	1	107.94	0.66	.419

**Table 5:** Generalized linear model with fixed effects of activity level, study phase, and shelter and their interactions predicting dogs’ activity.

**Source**	*Wald Chi-Square*	*df*	*p*
(Intercept)	44,164.95	1	<.001
Activity Level	8,087.86	4	<.001
Phase	0.94	2	.627
Shelter	40.35	1	<.001
Activity Level * Phase	179.83	8	<.001
Shelter * Activity Level	448.88	4	<.001
Shelter * Phase	3.47	2	.176
Activity Level * Phase * Shelter	43.35	8	<.001

A three-way interaction of activity level, phase, and shelter predicted the probability of activity across the five levels before, during, and after foster care at our two shelters (*p* < .001). Thus, the time dogs spent in Q1–Q5 activity differed, across study phases and between shelters. While these differences at the shelter level are notable, it is more approachable to describe the two-way interaction of activity level and phase on dogs’ activity in assessing the impact of weeklong fostering as a shelter welfare intervention.

**Figure 3:**
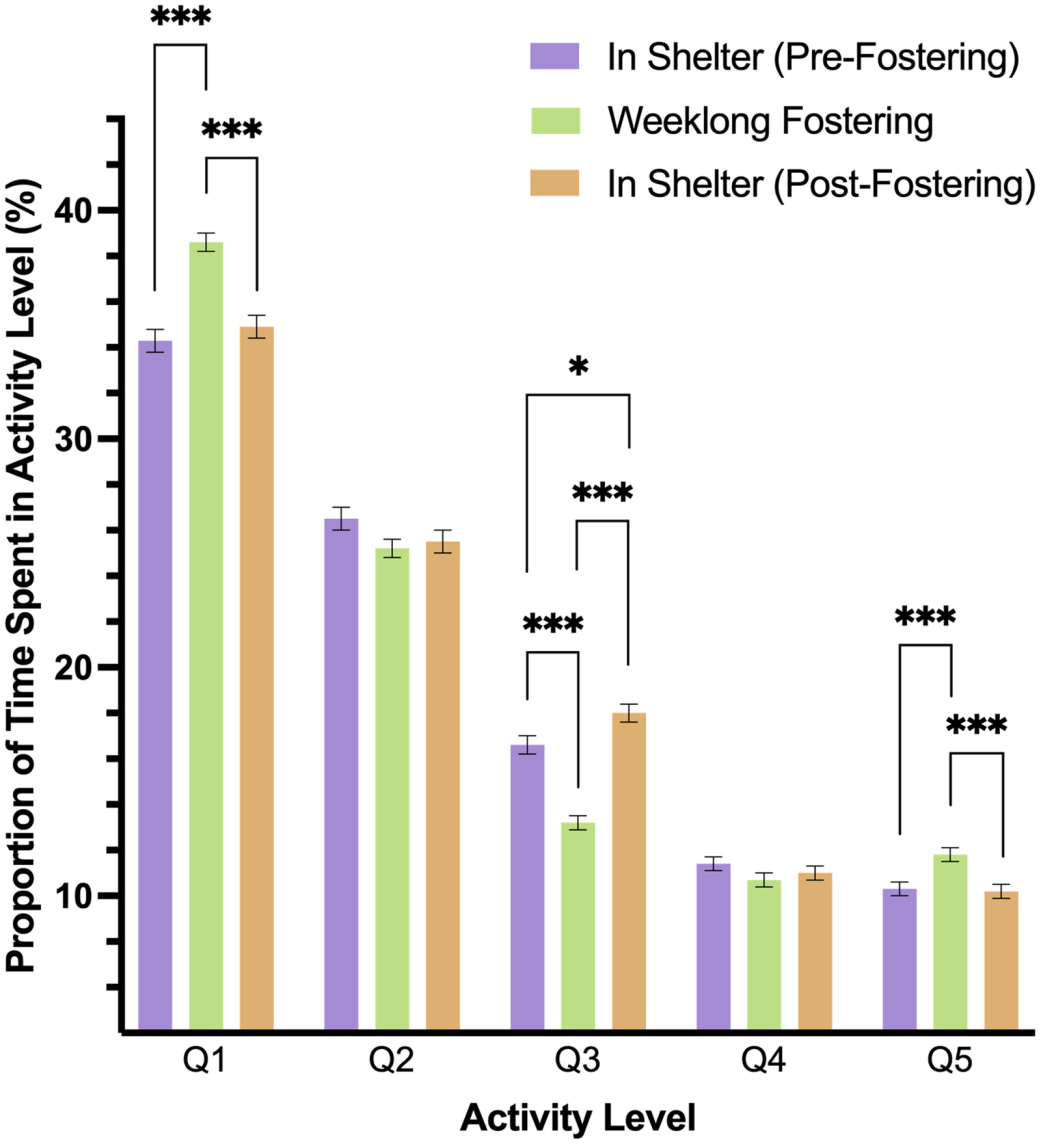
Estimated marginal means and standard errors of the proportion of time dogs spent in the five activity levels during the three phases of the study with Q1 representing the lowest level of activity, rest, and Q5, the highest activity. Note: * *p* = .05, *** *p* = < .001.

A two-way interaction of level and phase predicted dog’s activity in the study (*p* < .001). In pairwise comparisons, we found that dogs spent more time in Q1 (rest) and Q5 activity levels during fostering (Phase 2) than in either shelter phase (*p* < .001), but Q1 and Q5 activity did not differ between Phases 1 and 3 (Q1: *p* = .808; Q5: *p* = .998). Q3 activity differed across all study phases, such that this level of activity was lower during foster care than before or after in the shelter (*p* < .001). Moreover, Q3 activity was higher in Phase 3 as compared to Phase 1 (*p* = .035). Both Q2 and Q4 activity did not significantly differ during any phase of the study (Fig. 3).

### Co-housing: cortisol analyses

#### By day

Utilizing 210 cortisol:creatinine ratios from 42 dogs living at PACC, we analyzed these values across the initial five days of the study using a linear mixed model to detect an effect of housing, day, or a housing-by-day interaction with dogs’ sex, weight, age, and (log) LOS added as covariates. The final model, as determined by the smallest BIC value, is displayed in Table 6. Age was not retained. We detected no differences in dogs’ cortisol values across these days, by housing, or in an interaction of housing and day; thus, we did not find an effect of housing on dogs’ cortisol levels during these initial days.

In our second analysis, we investigated cortisol values during dogs’ five days in the animal shelter following foster care utilizing 195 cortisol:creatinine ratios. The final model, as determined by the smallest BIC value, is displayed in Table 6. As in the previous model, age was not retained. We found a housing-by-day interaction that was approaching significance (*p* = .089). Dogs’ length of stay (log-transformed) was significant in this model (*p* = .049), such that a 10% change in a dog’s length of stay is associated with a .14 change in its cortisol:creatinine ratio.

### Co-housing: activity analyses

#### By phase

To investigate the effect of housing on dogs’ activity in the shelter before and after fostering, we performed two generalized linear models using dogs’ time spent in Q1–Q5 activity in Phases 1 and 3. Across the five activity levels, 195 activity measurements (in hours) were included in each model with no dog variables entered as covariates. As with our analyses exploring the effects of housing on dogs’ cortisol levels, the first activity model utilized a binary variable to categorize dogs’ housing (*i.e.,* single or co-housed) while the second model included a three-level housing variable (*i.e.,* single, co-housed with a dog previously lived with, or co-housed with a new dog).

**Table 6:** Linear mixed models predicting dogs’ in-shelter cortisol values by housing type, day, and housing-by-day interaction, pre- and post-fostering.

**Source**	*Numerator df*	*Denominator df*	*F*	*p*
*In Shelter (Pre-Fostering)*				
Intercept	1	38.60	23.18	<.001
Housing	1	102.13	0.001	.977
Day	4	46.00	0.88	.481
Housing * Day	4	45.36	1.13	.354
Sex	1	37.51	0.10	.751
Weight (in kilograms)	1	37.11	1.54	.223
(log) LOS	1	49.65	2.09	.155
*In Shelter (Post-Fostering)*				
Intercept	1	32.80	4.21	.048
Housing	2	39.04	0.44	.646
Day	4	45.07	0.19	.943
Housing * Day	8	44.13	1.87	.089
Sex	1	32.68	0.78	.383
Weight (in kilograms)	1	32.28	3.51	.070
(log) LOS	1	33.05	4.17	.049

**Note.**

Dogs’ housing type in the shelter prior to fostering is living singly or co-housed; housing following fostering is either co-housed with a dog that was familiar or new to the dog or singly housed.

In our first model, we found an effect of activity level (*p* < .001), but no effect of housing type or an activity-level-by-housing interaction (Table 7). As expected, dogs’ activity significantly differed across the five levels, such that dogs spent the greatest proportion of time in rest (Q1) with decreasing amounts of time spent in Q2–Q5.

**Table 7:** Generalized linear models predicting dogs’ hours of activity in the shelter by housing type, activity level, and activity-level-by-housing interaction, pre- and post-fostering.

**Source**	*Wald Chi Square*	*df*	*p*
*In Shelter (Pre-Fostering)*			
(Intercept)	5,894.80	1	<.001
Activity Level	1,370.45	4	<.001
Housing	0.32	1	.964
Housing * Activity Level	7.35	4	.161
*In Shelter (Post-Fostering)*			
(Intercept)	5,395.35	1	<.001
Activity Level	1,368.64	4	<.001
Housing	4.68	2	.096
Housing * Activity Level	28.77	8	<.001

**Figure 4:**
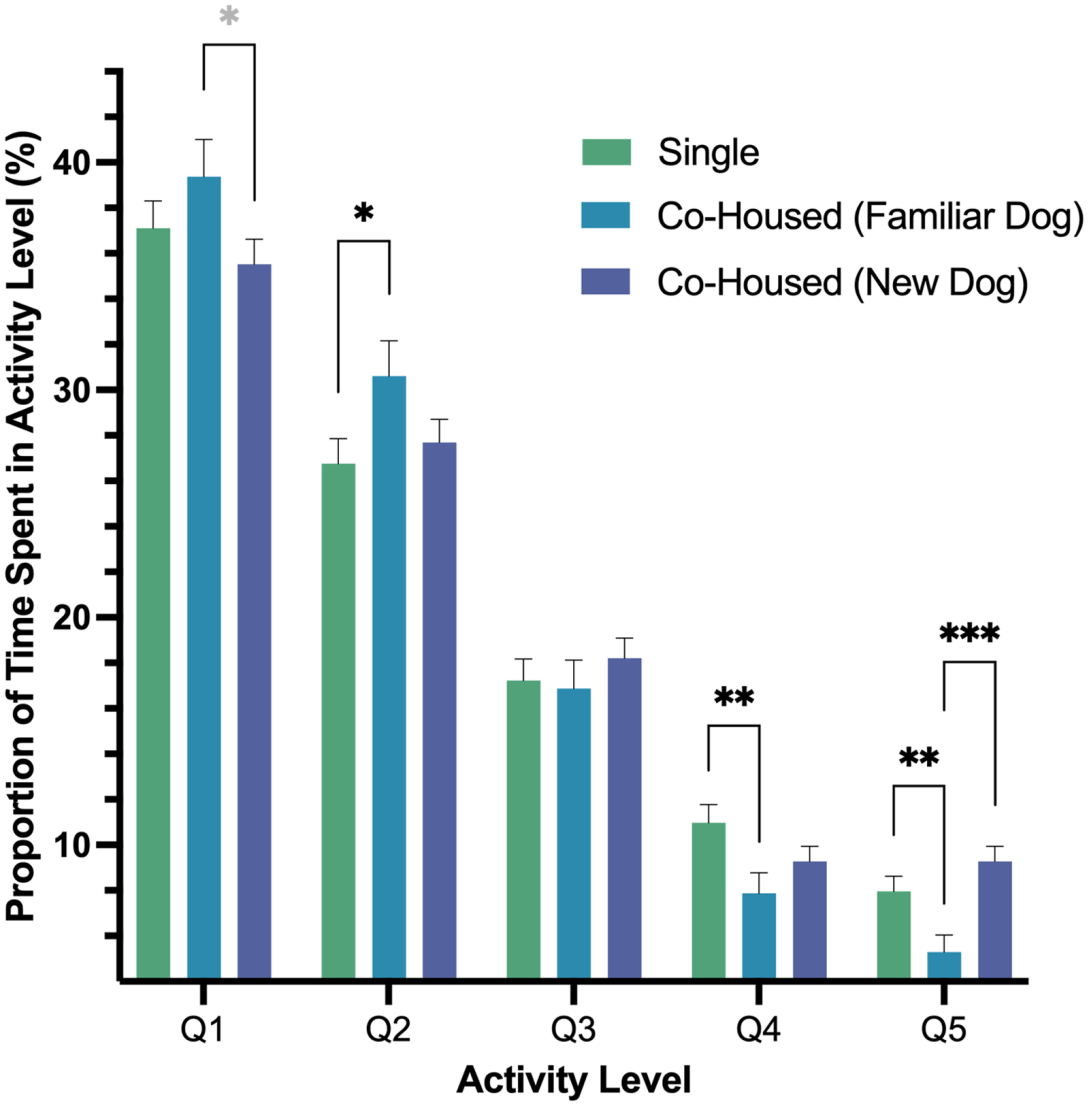
Estimated marginal means and standard errors of the proportion of time dogs spent in the five activity levels post-fostering in the shelter by housing type with Q1 representing the lowest level of activity, rest, and Q5, the highest activity. Note. * *p* = .051, * *p* = .05, ** *p* = .01, *** *p* = < .001.

In our second model, we found a significant interaction of housing and activity level (*p* < .001) as well as an effect of activity level (*p* < .001). In pairwise comparisons of the interaction, we found dogs’ Q1 activity, a measure of rest, was greater for dogs when they were co-housed with a dog that they were previously housed with prior to fostering *versus* dogs housed with a new dog, and that difference was nearly significant (*p* = .051). Q2 activity was higher when dogs were co-housed with a dog that they were previously housed with as compared to singly housing (*p* = .043). Moreover, we found that Q4 activity was lower when dogs were co-housed with a familiar dog *versus* being housed alone (*p* = .010). Lastly, the highest intensity activity, Q5, was lowest for dogs when they were housed with a familiar dog *versus* singly housed (*p* = .009) or being co-housed with a new dog (*p* < .001; Fig. 4).

## Discussion

In our investigation, we found that dogs’ C/C values were significantly lower during weeklong fostering as compared to the pre- and post-fostering phases in the shelter, with no significant difference between these in-shelter phases. Specifically, the intervention decreased dogs’ cortisol levels on Days 7–12 while in the caregiver’s home as compared to Days 1–5 (pre-fostering) and 13–17 (post-fostering) when dogs were living in the shelter. With regards to activity, dogs spent more time resting (Q1) and in the highest activity (Q5) while in the home than in either shelter phase, while Q3 activity was lowest during foster care and higher upon return to the animal shelter.

Thus, at both the individual (day) and aggregate (phase) levels, dogs’ cortisol and activity were improved by weeklong fostering. These findings align with previous studies that stays in a home (Fehringer, 2014; Gunter et al., 2019) aid the proximate welfare of dogs in animal shelters. While returning to the shelter is likely stressful for dogs, as indicated by changes in their cortisol, these levels are not higher than initial measurements taken in the shelter. Moreover, when we compare the magnitude of the treatment’s effect in this study to that of temporary fostering (1–2 nights, Gunter et al., 2019), we find a larger effect of seven days of fostering on dogs’ cortisol levels (*Temporary fostering d*_*av*_ = 0.4, *Weeklong fostering d*_*av*_ = 0.8; Cumming, 2012), indicating that dogs’ welfare was improved by these additional days in a caregiver’s home. Presently, it is unknown what the beneficial limits of fostering duration are for shelter-living dogs, and future investigations should explore longer lengths of foster care as well as post-fostering observation periods to further inform sheltering best practices.

Nevertheless, changes in cortisol, as a proxy for what an animal is experiencing, are difficult to interpret as stress itself is a complex physiological state (Ralph & Tilbrook, 2016); and it is the subjective appraisal of the environment by the individual, both in terms of its predictability and control over it, that describes the animal’s welfare (Veissier & Boissy, 2007). Decreases in cortisol as a result of an intervention are typically seen as welfare-enhancing, although prolonged exposure to stressors can lead to lower levels of cortisol (Hennessy, 2013). While fostering (overnight and longer) produces significant reductions in cortisol, we report the magnitude of these effects to better describe the scale of that difference (shelter and home) and aid in appreciating the practical consequence of interventions in an applied setting, particularly when the unit of measurement (cortisol) lacks intuitive meaning (Lakens, 2013).

In the current study, we found that the effect of shelter was comparable in magnitude to the intervention itself, suggesting that the environment is likely playing a role in these physiological differences and would align with shelter-level differences found in previous studies (Gunter et al., 2019; Gunter et al., 2021). While additive enrichment interventions have often been the subject of investigation in the animal shelter (Gunter & Feuerbacher, 2022), few studies have considered how environmental factors such as features of the kennel, density of dogs living in a single ward, husbandry that the dogs receive, and presence of people can influence their experience. In their study of shelter and owned dog activity, Hoffman, Ladha & Wilcox (2019) reported that the resting behavior of shelter dogs differed the most from owned dogs during the hours of 6 am to noon followed by noon to 6 pm when staff and potential adopters were likely present. Moreover, Hewison et al. (2014) found that prohibiting visitors during the afternoon at the shelter increased dogs’ sedentary behavior and reduced kennel noise, demonstrating an improvement in the dogs’ welfare.

In the absence of interventions that address the negative conditions present in most facilities (*e.g.*, noise, natural movement inhibition, and unpredictability), forgoing the shelter may be our best approach to improving welfare. The increase in rest observed in caregivers’ homes is likely associated with dogs’ lower cortisol levels. Conversely, the higher cortisol levels we observed in the shelter probably inhibited dogs’ rest, leading to more environmental alertness with such disruptions further compromising their welfare (Schork et al., 2022). Alongside improving dog’s physical environment and agency (*e.g.*, more movement and choice), foster care provides more behavioral interactions with people. Shelter dogs can experience greater attention, preferred foods, and other types of tactile reinforcers during their fostering stay. While these human-animal interactions are likely informed by caregiver abilities (*e.g.*, their knowledge about dog behavior), it is possible that the benefits of foster care might be further enhanced through training and support (Rault et al., 2020).

Under consideration in this study were the proximal effects of weeklong fostering on the welfare of shelter dogs; however, dogs’ distal welfare may be affected as well. Recently, Gunter et al. (2023) found that brief outings and temporary fostering stays increased dogs’ likelihood of adoption by five and 14 times, respectively, compared to dogs that did not receive these interventions. Additionally, we observed that temporary fostering caregivers adopted their dogs more often than individuals that provided brief outings. As such, it is possible that weeklong fostering could have further benefits for shelter dogs, including improving their adoption likelihood as well as being adopted by their foster caregivers. When we consider the longer duration of time dogs spent with their caregivers in this study, the distal effects of weeklong fostering may be even greater than those found with fostering interventions of shorter durations (Phillips & Gunter, 2024).

When examining the effects of single *versus* co-housing of shelter dogs, we found no differences in dogs’ cortisol values. Similarly, we found no effect of housing on dogs’ activity prior to fostering; however, we did detect a housing-by-level interaction with dogs’ activity in the shelter after weeklong fostering. When dogs were housed with a familiar dog rather than a new dog or being singly housed, dogs’ rest and Q2 activity was greater while their highest levels of activity, Q4 and Q5, were lower. While we failed to observe an impact of co-housing for dogs in the initial phase of our study, co-housing with a familiar dog upon re-entry to the shelter did positively affect the dogs’ behavior.

Across the literature, it is widely acknowledged that providing social animals the opportunity to interact with members of their own species plays an important role in positive welfare (Mellor, 2015). During stressful events, the presence of conspecifics may provide a type of social buffering, reducing the negative impact that these events have on the individual dog (Kikusui, Winslow & Mori, 2006). Prior research with kenneled and sheltered dogs has found that dogs display fewer behaviors associated with compromised welfare and more behavioral indicators of positive welfare when living together in groups or in pairs (Hubrecht, Serpell & Poole, 1992; Mertens & Unshelm, 1996; Dalla Villa et al., 2013) yet differences in cortisol have not been found when dogs have been co-housed (Grigg et al., 2017) or have been relatively small (Hecker et al., 2024).

When examining the effects of co-housing on the welfare of shelter-living dogs, the removal of a conspecific may have an adverse impact on the remaining dog that should also be considered. Walker, Waran & Phillips (2014) found that dogs that were co-housed in the shelter for an average of four months and then separated experienced negative changes in their behavior and physiology. Remaining dogs were more active (*e.g.*, running, circling, figure eights) and had increased immune function antibodies following separation from their familiar conspecific, suggesting that the loss was detrimental to their welfare. Taken together with the previous studies, our co-housing findings likely have implications for dogs returning to the animal shelter as well as those that are entering for the first time that were surrendered or found with another dog. When considering the stress of shelter entry (Hennessy et al., 1997), it may be advisable to house these dogs together, at least initially.

Practically, the effects of co-housing in the animal shelter may be most likely observed by changes in dogs’ behavior, although it is likely that the suitability of dogs that are co-housed together plays a role in the positive impacts observed in our study and that by Hecker et al. (2024). As such, dogs should be able to interact with each other, one-on-one, in larger areas prior to co-housing to reduce the likelihood of aggression once they are confined to a kennel. In the present study, dogs were positively impacted by a familiar dog following foster care; and changes in co-housed dogs’ behavior, more resting and less high activity, may be easier to observe than the more subtle behaviors observed by Hecker et al. (2024). However, it is unknown whether these differences in activity are perceptible without the use of technology; and as such, future research should consider the reliability of visual activity measurement in applied welfare assessment.

The monitoring of dozens or possibly hundreds of dogs compounds the difficulty of shelter welfare assessment. Resting (or lack thereof) most often occurs in the evenings and early mornings when staff are not in these facilities. Therefore, we propose considering wearable health and activity technologies (Yashari, Duncan & Duerr, 2015; Den Uijl et al., 2017; Rowlison de Ortiz et al., 2022) in animal sheltering to enable observation and recording as well as the potential for timely intervention across many individuals. If such technologies were utilized, dogs could be observed much more closely and behaviorally triaged with overnight or longer fostering interventions if they are resting for shorter periods of time or for smaller portions of a 24-hour period.

When considering the limitations in our study, it is possible that the differences in the dog populations at our two study sites contributed to our shelter-level variation in cortisol levels. Nevertheless, the discrepancies that could be identified often led to differences that were counter to our predictions. Dogs at CASPCA had lower but more varied cortisol levels, especially in the shelter prior to foster care, and were more often transferred into the shelter for placement than dogs at PACC. Previously, Herbel et al. (2020) found that one-and two-hour transports increased dogs’ cortisol levels, and these transport-related increases in cortisol continued to occur across multiple transport experiences. Yet recently, Hecker et al. (2024) found that dogs transported into the shelter for placement had lower cortisol levels than dogs surrendered by their owners. Dogs were also larger and had longer lengths of stay at PACC than CASPCA; but prior studies have found that dogs of greater weight and days in the shelter had lower, not higher, cortisol values (Gunter et al., 2019; Gunter et al., 2021); however, it is possible that the dogs’ older ages at PACC contributed to increased cortisol levels at the shelter level, as previously found (Gunter et al., 2019; Gunter et al., 2021), although no relationship between age and cortisol was detected in the present study. Lastly, as dogs that were overly aggressive or fearful of the collection tool were not enrolled in the study, our findings may not generalize to such individuals awaiting adoption in animal shelters.

## Conclusions

In the current investigation, C/C values were significantly lower, and dogs spent significantly more time resting during weeklong fostering as compared to phases in the shelter, both before and after foster care, with no significant differences between the two shelter phases. Our findings are in alignment with previous studies on canine fostering and extend our understanding about the benefits of foster care for shelter dogs’ proximate welfare. Future research exploring even longer durations of foster care as well as the distal effects of weeklong fostering would provide better comprehension of the impacts of human-animal interaction outside of the animal shelter. Practically, reducing the duration of time homeless dogs spend in the animal shelter through the utilization of foster homes may reduce animal care costs for these organizations.

Lastly, we found no evidence of an effect of co-housing on urinary cortisol or activity levels prior to fostering. However, when dogs returned to the animal shelter following foster care, their time in rest and lower activity increased and time spent in high activity decreased when housed with a familiar dog, suggesting a benefit to their welfare. It is possible that co-housing with a known dog upon reentry to the animal shelter provides a social buffering effect, improving their experience. As such, it is worth reconsidering the initial entry of dogs to the animal shelter and whether dogs that are surrendered by their owners or found together should be co-housed for better welfare.

##  Supplemental Information

10.7717/peerj.20608/supp-1 Supplemental Information 1 ARRIVE 2.0 checklist

## Additional Information and Declarations

### Data Availability

The following information was supplied regarding data availability: The data underlying this article are available at figshare: Gunter, Lisa; Feuerbacher, Erica (2025). The Implications of Weeklong Fostering and Co-Housing on Shelter Dog Welfare. University Libraries, Virginia Tech. Dataset. https://doi.org/10.7294/28764986.v1.
